# Estimating the Sex Gap in Depression-Free Life Expectancy Among Widowed Americans Aged 50 and Older: An Application Using the Interpolated Markov Chain Approach

**DOI:** 10.1177/08982643241233029

**Published:** 2024-02-21

**Authors:** Alessandro Feraldi, Cristina Giudici, Nicolas Brouard

**Affiliations:** 1 Research Group in Labor Demography, 9162 Max Planck Institute for Demographic Research, Rostock, Germany; 2 Department of Statistica Science, 210279 Sapienza University of Rome, Rome, Italy; 3 Department of Mortality Health and Epidemiology, 55859 French Institute for Demographic Studies, Paris, France

**Keywords:** widowhood, depression-free life expectancy, sex differences, multi-state life tables

## Abstract

**Objectives:**

Using Interpolated Markov Chain software, we compare the length of life with and without depression among married individuals and widowers, and the related sex differences.

**Methods:**

We applied a multi-state life table approach to estimate depression-free life expectancy among recent cohorts of older married and widowed women and men in the United States, using data from the Health and Retirement Study over a 7-year period (2012–2018).

**Results:**

The study revealed that the difference in life expectancy between sexes widens in the context of widowhood. At age 50, the sex gap in depression-free life expectancy is 0.8 years among married people, whereas the gap almost doubles to 1.7 years among widowed people.

**Discussion:**

By quantifying disparities in the duration of life affected by depression between married and widowed women and men, policymakers could properly allocate resources specifically to address the mental health needs of these groups.

## Introduction

Marital status can have significant effects on health. Numerous studies have found that being married is protective of health and is associated with lower morbidity and mortality, even when accounting for socio-economic and behavioral factors (Pijoan-Mas & Ríos-Rull, 2014; Rendall et al., 2011; Zueras et al., 2020). Indeed, a large body of literature has shown that married individuals have better physical and mental health and more social support than their unmarried counterparts (Ding et al., 2021; Rendall et al., 2011). Conversely, marital disruptions, such as widowhood, tend to contribute to poorer health and survival outcomes (Berntsen & Kravdal, 2012; Rendall et al., 2011). These disruptions can lead to decreased social support, changes in health behaviors, and increased psychological stress, which may, in turn, contribute to poorer health outcomes (Singham et al., 2021; Trivedi et al., 2009; Barrett, 2000).

The relationship between marriage and health may be explained by several factors. First, spouses can monitor each other’s health and health-related behaviors, promoting healthier lifestyles (Cockerham, 2005; Rendall et al., 2011). Furthermore, because married couples tend to have more family income, financial resources, and wealth than single people, they can purchase better medical care and a higher overall quality of life. Losing a spouse, particularly late in life, has been linked to a range of negative health outcomes, including poor mental health, decreased physical functioning, and increased risks of chronic disease and mortality (Ding et al., 2021; Rendall et al., 2011). Indeed, widowhood can be particularly challenging, as it often involves a significant loss of social support and a change in lifestyle (Singham et al., 2021).

While there is evidence that the benefits of marriage in terms of survival are more pronounced in men than in women, this male marriage advantage appears to narrow at older ages (Rendall et al., 2011). Among the theoretical explanations for this advantage are that traditional gender roles have historically assigned husbands the role of primary breadwinners, while wives were often responsible for domestic and caregiving duties: wives provide more social support and social integration to their husbands than husbands provide to their wives, and women are more likely than men to monitor health-promoting behavior among their spouses (Rendall et al., 2011). Conversely, husbands may be more likely than wives to provide socio-economic support. However, these roles have evolved significantly over time, and today, many couples have similar educational levels and share financial responsibilities and caregiving duties more equally or based on individual preferences and abilities (Kowalewska & Vitali, 2021).

Recent research has suggested that, on average, men are more likely than women to experience a decline in health following a marital disruption, such as widowhood. This is possibly due to gender disparities in access to social resources and support (Rendall et al., 2011). Additionally, marital disruption, including widowhood, has been associated with increased alcohol consumption, decreased body mass index in men, and a higher risk of smoking initiation/relapse in women (Ding et al., 2021; Rendall et al., 2011). In particular, widowhood has been linked to poorer physical and mental health, including increased risks of disease, disability, mortality (Rendall et al., 2011), and depression (Kristiansen et al., 2019a). Notably, research has shown that the experience of widowhood is closely connected to an increased vulnerability to depression (Bulloch et al., 2017; Ding et al., 2021; Ghesquiere et al., 2013; Rendall et al., 2011; Watkins & Waldron, 2017). Thus, measuring depression-free life expectancy (Dep-FLE) and its association with marital status is crucial in order to gain insights into a population’s overall health and well-being (Anstey et al., 2020; Huang et al., 2019; Matthews et al., 2009). By examining the psychological and emotional effects of widowhood, researchers can gain insights into how best to assist those individuals who are more susceptible to depression and other mental health conditions.

The present study is the first that compares estimates of Dep-FLE (namely, the number of years of life an individual can expect to live without depression) of widowed as against married people, among recent U.S. older adults, assessing gender-specific differences. This study employs the Interpolation of Markov Chain (IMaCh) software for the computation of multi-state life table (MSLT) approach: see further section 3 (Methodological considerations).

## Widowhood, Health, and Depression

As the population ages, the number of individuals who experience the loss of a spouse is likely to increase, particularly women compared to men, with the former often facing additional challenges related to financial security in widowhood. Understanding the impact of bereavement on individuals who have lost their spouse is critical to developing effective interventions and support systems. This is particularly true given an aging population and the gendered nature of widowhood (Ding et al., 2021; Rendall et al., 2011).

Among the various mental disorders, depression is a major health concern among the elderly, and the literature is consistent in recognizing a close connection between widowhood and an elevated susceptibility to depression (Abrams et al., 2019; Bulloch et al., 2017; Ding et al., 2021; Ghesquiere et al., 2013; Rendall et al., 2011; Watkins & Waldron, 2017). Depression is a severe mental illness with significant negative impacts on both an individual’s well-being and lifespan (WHO, 2017). It is closely linked to other physical health conditions like cardiovascular disease and diabetes, with increased risks of morbidity and mortality (Anstey et al., 2020; Freeman & Freeman, 2013; Huang et al., 2019; Matthews et al., 2009). Notably, the prevalence of depression and its impact may vary between genders, with studies suggesting a higher likelihood of women experiencing depression (Anstey et al., 2020; Freeman & Freeman, 2013). Systematic reviews and meta-analyses have shown that worldwide, the overall prevalence of depression is about 17.0%–20.0% among the widowed (Kristiansen et al., 2019a), compared to 8.5% among the more general population (Lim et al., 2018).

A recent systematic review and meta-analysis has shown that while the prevalence of depression is highest in the first months of widowhood, it remains elevated at least 5 years into widowhood (Blanner Kristiansen et al., 2019b). Furthermore, the literature on sex differences in depression in widowhood gives mixed results: some studies found that men are more likely than women to experience depression (Ding et al., 2021; Lee et al., 2001; Rendall et al., 2011; Trivedi et al., 2009); several others (Trivedi et al., 2009; Reddy et al., 2004) showed that widowhood has a greater adverse impact on the psychological well-being of women than of men, while still others found no sex differences at all (Li et al., 2005).

The literature on the relationships between marital status and depression provided estimates of the prevalence of depressive and major depressive symptoms among older adults according to marital status (Ding et al., 2021, for Australia; Li et al., 2005, for China; Copeland et al., 2004, for Europe; Reddy et al., 2004, India; Lee et al., 2001, the United States), or examined the factors associated with depression (Kristiansen et al., 2019a; Tucker et al., 2022). There is limited longitudinal research on how widowhood is associated with mental health expectancies, and especially with depression. In most of the studies focusing on healthy life expectancy (HLE), marital status is typically incorporated exclusively as a control variable (Giudici et al., 2019; Malhotra et al., 2021). To the best of our knowledge, only two studies investigated the link between marital status and HLE, using a longitudinal approach: one in the United States (Jia & Lubetkin, 2020) and one in Finland (Martikainen et al., 2014). The former investigated the impact of marital status on total life expectancy (TLE) and the active life expectancy of older adults, using the U.S. Medicare Health Outcome Survey. The latter examined life expectancy in long-term institutional care of older Finnish men and women. Both studies found a link between widowhood and lower HLE. Nevertheless, widowhood was not the subject of specific attention in either study.

Surprisingly, little is known about the dynamics of length of life with and without depression and its association with marital status. Therefore, further research is needed to quantify the Dep-FLE of women and men according to marital status and to fully understand the gender differences in the relationship between depression, HLE, and widowhood.

## Methodological Considerations

The most commonly used indicator to estimate the number of years of life an individual can expect to live in good health is the HLE. By combining data on mortality and morbidity (Beltran-Sanchez et al. 2015; Murray et al., 2015; Saito et al., 2014), this measure can support policymakers and public health officials in identifying high-risk groups and directing resources to reduce health disparities.

The existing research on HLE often focused on physical aspects of health, such as self-rated health and limitations in activities of daily living (ADLs and IADLs) (Pongiglione et al., 2015; Saito et al., 2014; Zaninotto et al., 2020). This is because these types of measures are relatively easy to quantify and are often used as indicators of overall health status. However, to have a more complete understanding of the association between HLE and marital status, it is important to consider cognitive and psychological well-being dimensions of health, notably depression (Anstey et al., 2020; Suthers et al., 2003; Ritchie et al., 1994).

Health expectancies (e.g., Dep-FLE) can be estimated using several methods, with the two most common approaches being the Sullivan and multi-state methods. The Sullivan method, introduced by Wolfbein (1949) and detailed by Sullivan (1971), combines health prevalence, for example, disability prevalence, from cross-sectional surveys with a period life table. However, it does not account for the incidence of disability in the reference period and may not adequately reflect current population health conditions. The multi-state method, pioneered by Rogers (1975) and further developed by Willekens (1979) and Brouard (1980, 2019), relies on longitudinal data to analyze transitions between health states, providing a period health expectancy that considers transitions between healthy and unhealthy states (in our study absence or presence of clinically significant depressive symptoms), while accounting for risk heterogeneity in different health status and population subgroups over time (e.g., the probability of dying depends on whether the individual experienced depression or not; additionally, married and widowed people may face different risks of depression, recovery, and mortality). Estimating health expectancies from longitudinal data involves the assumption that age-related health transitions adhere to a Markov process. There are several packages and software for the computation of the MSLT approach (Chungkham et al., 2023; Van den Hout et al., 2019). Among them, the Interpolation of Markov Chain (IMaCh) software, developed by Brouard and Lièvre, (Lièvre et al., 2003), has been employed in several analyses dealing with health (Lièvre et al., 2007; Crimmins et al., 2009; Andrade, 2010), including studies based on the Health and Retirement Study (HRS) (Chiu, 2019; Farina et al., 2020; Zimmer & Rubin, 2016), where information on health status is given by the interviews every 2 years. IMaCh offers the advantage of handling data from multiple waves and different intervals between surveys, accounting for varying survey interval lengths using interpolation. Additionally, it allows for estimating multiple transitions within an interval by specifying the time unit for interpolation (e.g., 1 year) and accommodates data from respondents who may have missed interviews between survey waves (Lièvre et al., 2003; Crimmins et al., 2009), which is frequent in longitudinal surveys. Finally, the program provides standard errors for life and health expectancy estimates, enabling the construction of confidence intervals for testing the statistical significance of differences between subgroups, such as marital status groups (Lièvre et al., 2003; Lièvre et al., 2007; Crimmins et al., 2009).

## Aim and Contribution

### Objective

The first aim of this study is to quantify Dep-FLE, life expectancy with depression (DepLE), and TLE among recent cohorts of older adults in the United States, while also examining how these indicators differ between married and widowed individuals. The second aim is to investigate whether there are any sex-specific differences in these indicators.

### Motivations

Unlike previous studies, this research examines the link between widowhood and Dep-FLE, which is rarely examined in the literature on HLE. Additionally, it employs the MSLT approach to measure Dep-FLE. This approach allows for more accurate estimations, compared to the Sullivan method (Jagger & Robine, 2011), and can be used to compare HLE among different subpopulations (in our case, sex-specific Dep-FLE in married and widowed individuals) without drawing on existing life tables. Furthermore, this study relies on IMaCh software to estimate the MSLT models, which offers significant advantages over recent hazard-based methods for estimating MSLTs (Crimmins et al., 2009).

By combining data on mortality and depression to estimate Dep-FLE in widowed and married women and men, the results of this study not only allow to identify group with higher risk of depression but also offer insights into the age-specific variation in the number of years that women and men in a different marital status can expect to live with or without depression. By quantifying the disparity in the duration of life affected by depression between married and widowed women and men, policymakers could properly allocate resources specifically to address the mental health needs of these groups. To our knowledge, this is the first study that uses a multi-state approach to estimate HLE pertaining to Dep-FLE in women and men, and according to marital status.

## Data and Methods

### Data Sources

Data were drawn from the RAND version of the HRS (Sonnega et al., 2014), which is a long-running survey of the health characteristics of older adults in the United States. The HRS has been collecting data from participants every 2 years since 1992 and has used exit interviews and mortality records to track participants’ vital status and date of death. The RAND version of the HRS is a cleaned and compiled version of the data that is user-friendly and easy to work with (Bugliari et al., 2020). The HRS is a nationally representative sample, which indicates that the study participants are representative of the broader population of older adults in the United States.

In order to estimate the prevalence, incidence, and transition to depression and recovery across waves, we used data on 17,852 married and widowed individuals interviewed from 2012 to 2018, with refreshment samples included at each wave.

We restricted our sample to 15,587 respondents aged 50 and older who had at least one observation during the follow-up and who remained married (12,048 individuals) or widowed (3539) during the follow-up period or until death. Respondents who had another marital status during the follow-up period (1349, e.g., separation or divorce) were excluded from the analyses. Since the model included marital status as a time-constant variable, respondents who remarried after widowhood (47 individuals) or who lost their spouse during the follow-up period (457 individuals) were also excluded. These individuals represented only a small portion of the total sample (approx. 3%). Finally, 412 individuals who had missing information on depression at the entry wave (less than 3%) were excluded from the analyses.

### Measurements

The Center for Epidemiologic Studies Depression (CESD-8) scale was adopted to measure depression in older adults. This scale is based on eight items that assess a range of symptoms associated with depression, including feelings of sadness, difficulty sleeping, and a lack of enjoyment in life (Niles et al., 2018; Karim et al., 2015). The CESD-8 scale is scored by summing the responses to the eight items, with a cut-off score of three being used to indicate the presence of clinically significant depressive symptoms. This cut-off score has been validated in previous research and has been shown to be effective in identifying older adults with clinically relevant levels of depression. In terms of marital status, respondents were categorized as married if they remained married during the follow-up period or until death or as widowed if they remained widowed during the follow-up period or until death.

### Statistical Analysis

**Figure 1.**
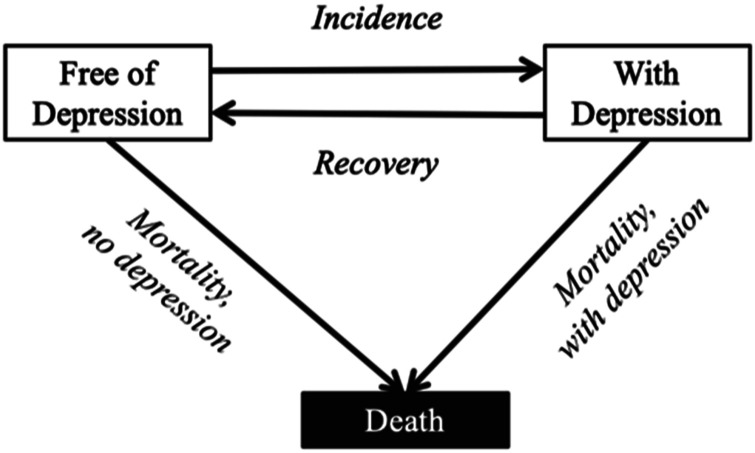
Structure of a multi-state life table model with two health states. The transitions are age-dependent and conditioned by health status. Marital status is used as a covariate by excluding those whose marital status changed in the follow-up.

A MSLT approach was used to estimate the age-specific hazard rates of transitions to depression, recovery, and death (see Figure 1). The MSLT approach is a method for modeling the movement of individuals between different states over time. In this type of model, individuals can move between a finite number of states and can also exit and re-enter the same state. This method is often used to study the progression of diseases or other health conditions that involve changes in an individual’s health status over time. It is based on the assumptions of the Markov process, which states that the probability of transitioning from one state to another depends only on the current state and the time elapsed and is not influenced by the previous states or the path taken to reach the current state (Hougaard, 1999). Analyses were carried out using the Interpolated Markov Chain (IMaCh) software version 0.99r43 (Brouard, 2019; Lievre et al., 2003). This technique partitioned the time intervals between consecutive interviews into shorter steps and then modeled the resulting transition probabilities by multinomial logistic regression on age and covariates. In particular, our model included three possible states: no depression (coded as 1), depression (coded as 2), and death (coded as 3). Accordingly, transition probabilities were estimated based on a series of 3 × 3 matrices:

$${hP}_{x}=\left(\begin{array}{lll} {hp}_{x}^{11} & {hp}_{x}^{12} & hp_{x}^{13}\\ {hp}_{x}^{21} & hp_{x}^{22} & {hp}_{x}^{23}\\ 0 & 0 & 1 \end{array}\right)$$

where ${hp}_{x}^{ij}$ indicated the probability for an individual with age *x*, observed in state *i* at the first interview, to find him/herself at the next interview and age *x+h*, in state *j*. But as exposure time *h* was not the same for all individuals (some interviews were missing for one or more waves, but not for a subsequent wave), the Interpolated Markov Chain procedure consisted of estimating a multinomial logistic (Eq. (1)) on a specific duration *h* that was lower than or equal to the mean interval between two waves. As the interval between two successful interviews was not necessarily a multiple of the elementary unit *h*, the likelihood was then interpolated within boundaries. Ages and dates of interviews were expressed in months.

Marital status (coded *z*) was incorporated into the model as a covariate to highlight heterogeneities in the changes of depression status over time between married and widowed people:

(1) $$\ln\frac{p_{x}^{ij}}{p_{x}^{ii}}=\alpha_{ij}+\beta_{ij}\;x+\gamma_{ij}\;z$$

Estimated transition rates were then applied to a synthetic cohort to compute the health expectancy for people either affected by or free from depression at the initial age (Hougaard, 1999). Accordingly, we were able to create a MSLT that could be used to estimate the Dep-FLE, DepLE, and TLE for the study population.

The IMaCh software provided standard errors for the estimated parameters, which allowed us to derive standard errors for the life expectancies implied in the transition probabilities. This was an important characteristic that enabled us to assess whether the results were statistically meaningful. Analyses were performed separately for men and women. The sex gap in life expectancy was computed as the difference in total and in health-specific life expectancy (no depression and depression) between females and males (i.e., life expectancy of females minus life expectancy of males).

## Results

The sample includes 8516 women (54.6%) with a mean age of 67.8 years and 7071 men (45.3%) with a mean age of 66.7 years. Overall, during the follow-up, respectively, 1342 (17.9%) and 1521 (20.0%) women and men died. More specifically, among men, 1023 out of 6420 (15.9%) married individuals and 319 out of 651 widowed individuals (49.0%) died during the follow-up; among women, 522 out of 5628 (9.0%) married individuals and 999 out of 2888 widowed individuals (36.0%) died during the follow-up. As mortality is lower for women compared to men, it is not surprising to find a larger share of widowed women (33.9%) than of men (9.1%). We also found that the overall prevalence of depression is more important in women (21.5%) compared to men (15.0%), and in widowed (27.4%) than in married (15.9%) people. Notably, while the prevalence of depression is significantly higher in married women than in married men (respectively, 18.5% and 13.7%), it is similar among widowed women and men (respectively, 27.4% and 27.8%) (Table 1).

**Table 1.** Prevalence of Depression and Widowhood by Sex (* Denote Statistically Significant Differences).

	Total Sample		Women		Men		Sex Comparison
	*N*	%	*N*	%	*N*	%	
Depression	2889	18.5	1829	21.5	1060	15.0	*
Widowed	3539	22.7	2888	33.9	651	9.2	*
Depression in married	1918	15.9	1039	18.5	879	13.7	*
Depression in widowed	971	27.4	790	27.4	181	27.8	
Overall	15,587		8516	54.6	7071	45.4	*

**Figure 2.**
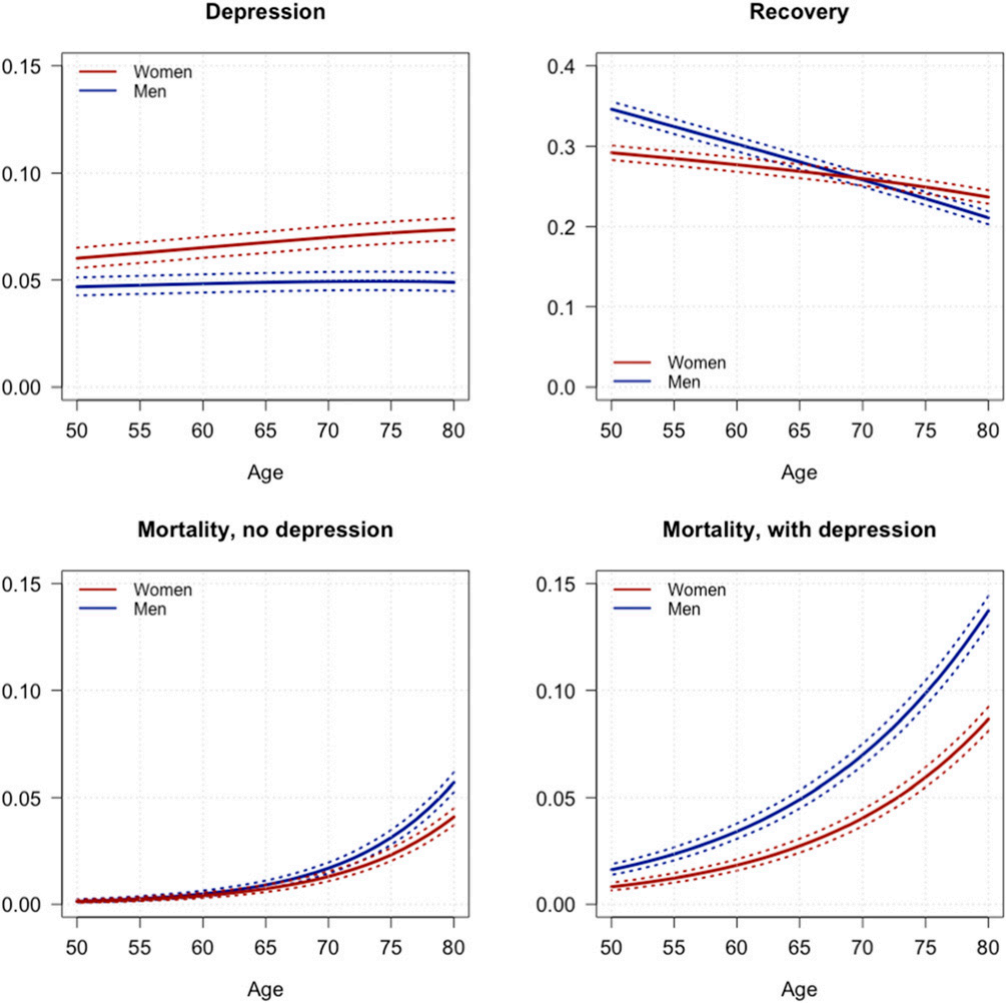
Annual probabilities to enter depression by age and sex, as well as for recovering from depression and competing probabilities to die for depressed and non-depressed individuals (with a 95% confidence interval).

Figure 2 shows the transition probabilities per year from different initial states of health. As expected, the probability of dying is higher among people with depression than among people free from depression and is higher among men than among women. The probability of dying is closer among women and men who are not affected by depression, with small differences at older ages (70+ years). Among people affected by depression, the probability of dying is significantly higher among men than among women at all ages.

The annual incidence of depression at age 50 (which is very similar in magnitude to the annual probability) is higher in women than in men at any age. The incidence of recovery is slightly but significantly higher in men than in women at younger ages (until age 65), and decreases with age in both women and men, with a lower slope for women. Similar incidence levels are observed at older ages in both sexes (Figure 2). The age-specific probabilities described above have been applied to a fictitious cohort of each initial state to compute life expectancy free from depression (Dep-FLE), as well as with depression (Dep-LE), at each age. By weighting each status-based probability of death by the proportion of people in each status based on the observed HRS prevalence, we obtained the total mortality rates.

Table 2 shows the estimates, 95% confidence intervals, and proportions of Dep-FLE and DepLE across ages for women and men. At age 50, the average number of years women and men expect to live without depression is similar, at respectively 26.3 years (25.7–26.9) and 25.9 years (25.2–26.6). At older ages (i.e., 70+), significant sex differences in Dep-FLE are observed. The proportions of remaining life free from depression are similar across ages in both sexes and are slightly larger in men than in women: between ages 50 and 80, the proportion ranges are 78.1–80.9% in women and 84.7–86.8% in men. Conversely, the proportion of remaining life with depression is larger in women than in men, particularly at younger ages. At age 50, a woman can expect to live on average 6.2 years with depression, compared to only 3.9 years for a man. The sex absolute gap (females − males) in DepLE decreases with age, ranging from 2.3 years at age 50 to 0.9 years at age 80.

**Table 2.** Depression-Free Life Expectancy (Dep-FLE), Proportion, and Life Expectancy With Depression (DepLE) Across Ages, by Sex (95% Confidence Intervals in Parentheses, * Denote Statistically Significant Differences).

Age	Women			Men			Sex Gap (F-M)	
	Dep-FLE	%	DepLE	Dep-FLE	%	DepLE	Dep-FLE	DepLE
50	26.3 (25.7–26.9)	80.9	6.2 (5.9–6.6)	25.9 (25.2–26.6)	86.8	3.9 (3.7–4.2)	0.4	2.3*
55	22.5 (21.9–23.0)	80.4	5.5 (5.2–5.8)	21.9 (21.3–22.5)	86.4	3.4 (3.2–3.7)	0.6	2.1*
60	18.9 (18.4–19.4)	80.0	4.7 (4.5–5.0)	18.2 (17.7–18.7)	86.1	2.9 (2.7–3.1)	0.7	1.8*
65	15.5 (15.0–15.9)	79.6	4.0 (3.8–4.2)	14.7 (14.2–15.1)	85.8	2.4 (2.2–2.6)	0.8	1.6*
70	12.4 (12.0–12.8)	79.1	3.3 (3.1–3.5)	11.5 (11.1–11.9)	85.4	2.0 (1.8–2.1)	0.9*	1.3*
75	9.6 (9.3–10.0)	78.6	2.6 (2.4–2.8)	8.7 (8.4–9.1)	85.1	1.5 (1.4–1.7)	0.9*	1.1*
80	7.2 (6.9–7.6)	78.1	2.0 (1.8–2.2)	6.4 (6.1–6.7)	84.7	1.1 (1.0–1.3)	0.8*	0.9*

### Depression-Free Life Expectancy by Sex and Marital Status

In Table 3, we present estimates of TLE, Dep-FLE, and DepLE by sex and marital status, at every 5 years from age 50 to age 80. To further investigate variations in life expectancies by marital status, we calculated the differences in TLE, Dep-FLE, and DepLE between widowed and married individuals. Overall, both widowed women and widowed men have considerably shorter absolute and relative Dep-FLE than married people. A stronger association is observed between marital status and Dep-FLE than between marital status and TLE or DepLE.

**Table 3.** Total Life Expectancy (TLE), Depression-Free Life Expectancy (Dep-FLE), Life Expectancy With Depression (DepLE), and Absolute Differences Between Widowed (W) and Married (M) Individuals Across Ages, by Sex (95% Confidence Intervals in Parentheses, * Denote Statistically Significant Differences).

	Women			Men		
Age	Married (M)	Widowed (W)	W − M	Married (M)	Widowed (W)	W − M
*Total Life Expectancy—TLE (95% CI)*						
50	32.5 (31.7–33.3)	32.2 (31.2–33.2)	−0.3	30.1 (29.4–30.7)	27.7 (26.2–29.3)	−2.3*
55	27.9 (27.1–28.7)	27.7 (26.8–28.6)	−0.3	25.5 (24.9–26.1)	23.4 (21.9–24.8)	−2.2*
60	23.5 (22.8–24.3)	23.3 (22.5–24.1)	−0.2	21.2 (20.7–21.8)	19.2 (18.0–20.5)	−2.0*
65	19.4 (18.6–20.2)	19.2 (18.6–19.9)	−0.2	17.2 (16.7–17.7)	15.4 (14.4–16.5)	−1.8*
70	15.6 (14.9–16.3)	15.5 (14.9–16.0)	−0.1	13.6 (13.1–14.0)	12.0 (11.1–12.9)	−1.6*
75	12.2 (11.5–12.9)	12.1 (11.6–12.6)	−0.1	10.4 (10.0–10.8)	9.1 (8.4–9.8)	−1.3*
80	9.2 (8.6–9.9)	9.2 (8.8–9.6)	0.0	7.7 (7.3–8.1)	6.7 (6.1–7.2)	−1.0*
*Depression-Free Life Expectancy—Dep-FLE (95% CI)*						
50	26.9 (26.1–27.7)	23.9 (22.7–25.1)	−3.0*	26.2 (25.5–26.9)	22.2 (20.3–24.1)	−4.0*
55	23.2 (22.4–23.9)	20.6 (19.6–21.6)	−2.6*	22.2 (21.6–22.8)	18.6 (17.0–20.3)	−3.6*
60	19.5 (18.8–20.3)	17.4 (16.5–18.2)	−2.2*	18.4 (17.9–19.0)	15.3 (13.9–16.7)	−3.1*
65	16.1 (15.4–16.8)	14.4 (13.7–15.0)	−1.8*	14.9 (14.4–15.4)	12.2 (11.0–13.4)	−2.7*
70	13.0 (12.3–13.7)	11.6 (11–12.1)	−1.4*	11.8 (11.3–12.2)	9.5 (8.5–10.4)	−2.3*
75	10.2 (9.5–10.8)	9.1 (8.6–9.5)	−1.1	9.0 (8.6–9.4)	7.2 (6.4–7.9)	−1.8*
80	7.7 (7.1–8.3)	6.9 (6.5–7.3)	−0.8	6.7 (6.3–7.0)	5.3 (4.7–5.8)	−1.4*
*Life Expectancy with Depression—DepLE (95% CI)*						
50	5.6 (5.2–6.0)	8.3 (7.5–9.1)	2.7*	3.9 (3.6–4.2)	5.6 (4.4–6.7)	1.7*
55	4.8 (4.4–5.1)	7.1 (6.4–7.8)	2.3*	3.3 (3.1–3.6)	4.7 (3.8–5.7)	1.4*
60	4.0 (3.7–4.3)	6.0 (5.4–6.5)	2.0*	2.8 (2.6–3.0)	4.0 (3.2–4.7)	1.1*
65	3.3 (3.0–3.6)	4.9 (4.5–5.3)	1.6*	2.3 (2.1–2.5)	3.2 (2.6–3.9)	0.9*
70	2.6 (2.4–2.9)	3.9 (3.6–4.2)	1.3*	1.8 (1.7–2.0)	2.6 (2.0–3.1)	0.7
75	2.0 (1.8–2.3)	3.0 (2.8–3.3)	1.0*	1.4 (1.3–1.6)	2.0 (1.5–2.4)	0.5
80	1.5 (1.3–1.7)	2.3 (2.1–2.5)	0.8*	1.1 (0.9–1.2)	1.5 (1.1–1.8)	0.4

Widowhood is significantly associated with shorter TLE only among men (Table 3). Married and widowed women have similar TLE at all ages. By contrast, at age 50, the average life expectancy of widowed men is 2.3 years shorter than that of married men. The TLE absolute gap between widowed and married men decreases with age, but it is still significant at age 80.

The study also shows that the difference in life expectancy between the sexes widens in the context of widowhood. The 2.4-year gap in life expectancy at age 50 between married women and men increases to 4.5 years among widowed people. Both widowed men and women can expect to live fewer years without depression than their married counterparts: in particular, at age 50, Dep-FLE is 3.0 years lower among widowed women and is 4.0 years lower among widowed men. The absolute gap in Dep-FLE between widowed and married individuals narrows with age, becoming insignificant for women after age 70, but remaining significant for men across all age groups. Compared to their married counterparts, widowed women have longer DepLE at all ages, ranging from 2.7 years at age 50 to 0.8 years at age 80. Among men, DepLE in widowed individuals is significantly longer than in their married counterparts only at the earlier stages, specifically between the ages of 50 and 65.

To provide a more in-depth analysis of the variations in the sex differences in the length of life free from depression according to marital status, we have looked at differences in Dep-FLE between married and widowed men and women (see Table 4). The results show that widowhood is significantly associated with a larger Dep-FLE disadvantage for men at all ages: at ages 50–80, the sex gap in Dep-FLE ranges from 0.8 to 1.2 years among married people; whereas among widowed people, this gap almost doubles, ranging from 1.7 to 2.1 years.

**Table 4.** Sex Gap in Dep-FLE (Females Minus Males) Across Ages, by Marital Status.

Age	Married (M)	Widowed (W)
50	0.8 (0.6–0.9)	1.7 (1.0–2.4)
55	1.0 (0.8–1.1)	1.9 (1.3–2.6)
60	1.1 (0.9–1.3)	2.1 (1.5–2.7)
65	1.2 (1.0–1.4)	2.1 (1.7–2.6)
70	1.2 (1.0–1.5)	2.1 (1.7–2.5)
75	1.2 (0.9–1.4)	1.9 (1.6–2.2)
80	1.1 (0.8–1.3)	1.7 (1.5–1.9)

**Figure 3.**
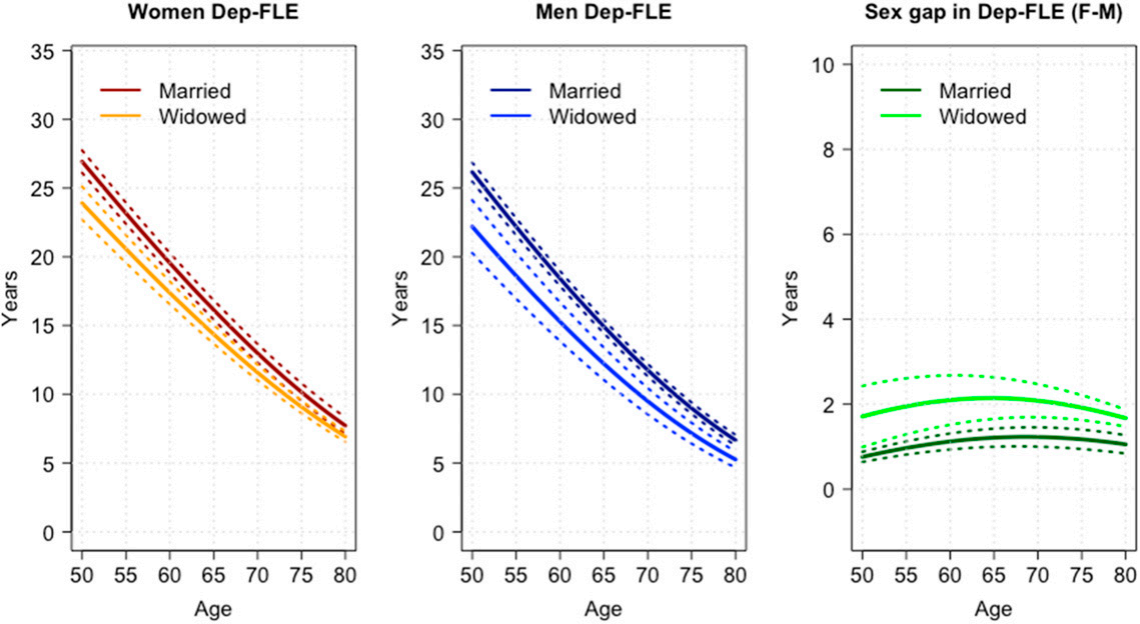
Depression-free life expectancy (Dep-FLE) by sex and marital status and sex gap (females (F) minus males (M)) by marital status, across ages.

The extent of the gap in Dep-FLE between widowed and married people, and between men and women, is displayed in Figure 3. The figure, which shows Dep-FLE across ages, by sex and marital status, clearly indicates that the sex gap in Dep-FLE is larger for widowed than for married people.

## Discussion

This study provides population-based estimates of Dep-FLE among older Americans, examining how it differs by sex and by marital status. Results show that, on average, women spend more years of their life than men living with depression, and the absolute sex differences are larger at younger than at older ages. Indeed, at age 50, DepLE was found to be 2.3 years higher in women than in men. This finding corresponds to the widely reported higher prevalence of depression among women. Moreover, our study offers insights into the differences in Dep-FLE between married and widowed individuals, showing that widowed women and men have a shorter Dep-FLE than their married counterparts. The finding that females live more years with depression than males is in line with the literature on what has been termed the female–male health-survival paradox (Oksuzyan et al., 2010), which is generally based on other common health indicators (e.g., disability, chronic conditions) (Apinonkul et al., 2015). A notable finding of our study is that the years spent with depression appear to be relatively constant across older ages. This observation is consistent with the results of other studies, which found that the expected length of life with cognitive impairment and mental disorders (including depression) tends to be relatively constant across older ages (Apinonkul et al., 2015; Brugha et al., 2013; Suthers et al., 2003).

### The Association Between Widowhood and Depression-Free Life Expectancy

Our analysis also uncovered a strong association between widowhood and Dep-FLE: compared to their married counterparts, Dep-FLE at age 50 was found to be 3.0 years lower among widowed women and 4.0 years lower among widowed men. Several studies have found that, compared to married people, widows and widowers have an increased risk of mental illness, and particularly of depression and anxiety (Ding et al., 2021; Trivedi et al., 2009). This risk is particularly high in the immediate aftermath of a spouse’s death, when the widowed individual is most likely to experience intense feelings of grief and loss. For example, a study by Trivedi et al. (2009) found that the prevalence of these symptoms was highest in the first 6 months following the death of a spouse and, then, gradually declined over time.

Globally, our study found that widowed people face the double burden of shorter overall life expectancy and longer DepLE compared to married individuals. Research indicates that, generally, the loss of intimate relationships, especially through death, negatively affects health and well-being. Bereavement research, which concentrates on the deaths of close family members, unequivocally demonstrates that losing a family member can have profound physiological consequences and may heighten the risk of mortality. Within the bereavement literature, the death of a spouse, particularly when it is unexpected, premature, and violent, stands out as one of the most traumatic events in the course of an individual’s life (Bolano & Arpino, 2020; Umberson, 2017).

Whether widowhood is a more difficult psychological experience for men or for women is still an open debate. On the one hand, widowhood might be associated to greater financial challenges for women than for men, which could lead to lower psychological well-being among widows. On the other hand, marriage may be more psychologically detrimental for men than for women, as men seem to have more to lose than women from the death of a spouse in terms of social resources and support (Brown and Lee, 2017; Ding et al., 2021; Rendall et al., 2011). The results of this study tend to support the latter observation. The women in our sample are more likely to be widowed than the men. Although we found similar levels of depression in widowed women and men, we also observed that the differences in depression prevalence between widowed and married individuals are significantly larger among men than among women. Analyzing the causal link between widowhood and depression was not the purpose of our study. However, we can speculate that widowhood substantially contributes to the increase in the Dep-FLE disadvantage of men at all ages between 50 and 80 years.

The finding that the absolute sex gap in Dep-FLE is larger among widowed than among married individuals may be partially explained by sociological factors related to gender differences in the health benefits of marriage and caregiving responsibilities. Research has consistently shown that marriage has numerous benefits for both men and women, including better physical health, more social support, and reduced mortality rates (Ding et al., 2021; Rendall et al., 2011). However, it appears that the health benefits of marriage are larger for men than for women (Ding et al., 2021; Brown and Lee, 2017). This pattern is commonly attributed to men being more likely than women to rely on their spouse for emotional and social support, as well as for instrumental benefits, such as better domestic and care support. Indeed, women typically provide more spousal care than men (Revenson et al., 2016; Swinkels et al., 2019). In addition, women are more likely than men to have larger social networks and sources of support outside of marriage (Cornwell, 2011). Consequently, widowed men may be less prepared for the loss of their spouse and have fewer social supports than widowed women, who are usually better connected to family and friends. Therefore, when a man loses his spouse, he may suffer a greater overall psychological decline. This lack of support could contribute to greater psychological distress and lower Dep-FLE among widowed men than among widowed women, leading to a larger sex gap in Dep-FLE in widowed than in married individuals.

The different association between widowhood and depression in women and men suggests a need for gender-specific policies to promote mental well-being. Despite women being the primary recipients of widowhood policies due to their longer life expectancy, there is the risk of underestimating the mental health needs of widowed men. Findings suggest a need to reassess resource allocation to effectively address these challenges. To achieve this, policymakers should consider adopting a comprehensive approach that considers both gender and marital status, to develop more effective strategies to promote mental health, prevent depression, and enhance the overall well-being of older adults. For example, the creation of support programs specifically tailored to widowed individuals, encompassing counselling services, support groups, and community engagement initiatives, can help to reduce the psychological challenges they face, particularly in the immediate aftermath of losing a spouse. Given that women are more likely than men to become widowed, the implementation of public health campaigns and interventions aimed at increasing overall life expectancy, with a specific focus on factors contributing to higher mortality rates in men, could diminish the risk of women experiencing widowhood. Moreover, recognizing the potential financial challenges encountered by the widowed, especially women, policies might prioritize financial support to ensure economic stability and reduce stressors that can contribute to mental health issues. Nonetheless, encouraging the development of broader social networks for the widowed, especially men, may significantly contribute to mitigating the psychological impact of losing a spouse.

Finally, the paper analyzed data up to 2018, near the onset of the COVID-19 pandemic. COVID-19 has disproportionately affected the elderly, leading to a higher mortality rate among this demographic group (Wang et al., 2022; Yanez et al., 2020). U.S. life expectancy experienced a decline in the years 2019–2021, partially offset in the period 2021–2022 (Arias et al., 2023). Although these issues were out of our study’s purpose, we can speculate that these factors could reinforce the study’s findings using more recent data. The increased mortality rates among the elderly due to COVID-19 may contribute to higher levels of depression, given the potential associated stress of relationship losses (Wang et al., 2022). The pandemic also induced social isolation and loneliness, particularly among older individuals. This may further worsen mental health issues, potentially influencing marital status and its association with Dep-FLE. Moreover, disruptions in health care access during the pandemic could pose additional challenges for individuals seeking mental health care. Nevertheless, the ways in which health expectancies will be influenced in the long term by COVID-19 pandemic have not been determined yet (Spiers et al., 2021). Further research addressing these factors is needed to provide a more nuanced understanding of the dynamics between depression, marital status, and overall well-being among older individuals in the future.

### Strengths and Limitations

To test the robustness of our findings, a sensitivity analysis was conducted. The same analyses were carried out on the sample stratified according to whether they were White or non-White (i.e., Black, Hispanic, and others) and their level of education (i.e., lower education: less than a high school degree, a high school degree, or general education development; and higher educated: some college or a college degree or higher). We found similar associations between widowhood and Dep-FLE and similar sex differences in Dep-FLE in each of these subgroups. We conducted additional sensitivity analyses and robustness checks by individually excluding feelings of sadness and difficulty sleeping from our analysis. The results suggested that the differences in Dep-FLE are not statistically significant when we exclude feelings of sadness or difficulty sleeping from the scale.

A limitation of this study is that depression was measured based on self-reported symptoms. This measure is subject to bias, as individuals may not accurately report their symptoms due to either a lack of awareness of their condition or because of the stigma associated with mental illness. Additionally, some individuals may not want to share their depression because they consider it a private affair, which can lead to an underestimation of the real prevalence of depression. Another limitation is that the depression scale used in this study (the CESD-8 scale) only includes a few items. While the CESD-8 scale has been shown to be effective in detecting depression, especially among older adults (Niles et al., 2018), there are other possible dimensions of depression that may provide different scales. Nonetheless, we decided to use this scale because of evidence showing that the CESD-8 scale is a short, valid, and reliable measure of depression that is acceptable for use among community-dwelling respondents and is not substantially influenced by conditions during household interviews (Briggs et al., 2018; Fountoulakis et al., 2001; Niles et al., 2018). Third, it is beyond the scope of the analysis to determine the causality of the relationships between widowhood, depression, and life expectancy with and without depression.

Despite these limitations, this study provides, for the first time, Dep-FLE estimates for U.S. women and men, and according to marital status. The MSLT approach allowed us to generate accurate estimates based on the incidence of depression in the reference period. Moreover, the analysis was conducted using a representative sample of U.S. adults aged 50 and older. Estimates of Dep-FLE are extremely helpful for researchers and policymakers seeking to develop new policies and interventions and to evaluate and adjust existing social and medical policies and programs. As the population ages, it is important for older people to maintain their mental and physical health, not only for their own benefit but also to reduce strain on health care systems and the economy (Brown and Lee, 2017; Ding et al., 2021; Rendall et al., 2011).

### Conclusion

The study reveals that the life expectancy sex gap increases with widowhood. While the TLE of women at age 50 is not affected by widowhood, widowed men are at a higher risk of dying. This increased gap can be explained by a longer period of depression among women in widowhood, compared to their male counterparts. These results confirm the importance of taking marital status into account in the development of policies and interventions aimed at supporting the health and well-being of individuals. Focusing greater attention on depression, particularly in widowed individuals, could help in the early detection of associated symptoms and ultimately reduce the complications associated with the disease.
